# Body Fat and Obesity Rates, Cardiovascular Fitness, and the Feasibility of a Low-Intensity Non–Weight-Centric Educational Intervention Among Late Adolescents: Quasi-Experimental Study

**DOI:** 10.2196/67213

**Published:** 2025-01-24

**Authors:** Areeg Zuair, Fahad M Alhowaymel, Rola A Jalloun, Naif S Alzahrani, Khalid H Almasoud, Majdi H Alharbi, Rayan K Alnawwar, Mohammed N Alluhaibi, Rawan S Alharbi, Fatima M Aljohan, Bandar N Alhumaidi, Mohammad A Alahmadi

**Affiliations:** 1 Department of Community Health Nursing, Taibah University, Janadah Bin Umayyah Road, Medina, 42353, Saudi Arabia, 966 594800400; 2 Department of Nursing Science, Shaqra University, Shaqra, Saudi Arabia; 3 Department of Nutrition and Food Science, Taibah University, Medina, Saudi Arabia; 4 Department of Medical Surgical Nursing, Taibah University, Medina, Saudi Arabia; 5 Department of Sport Science and Physical Activity, Taibah University, Medina, Saudi Arabia; 6 Department of Nursing, Prince Mohammed Bin Abdulaziz Hospital, Medina, Saudi Arabia; 7 Department of Oncology, King Salman Bin Abdulaziz Medical City, Medina, Saudi Arabia; 8 Emergency Department, King Salman Bin Abdulaziz Medical City, Medina, Saudi Arabia

**Keywords:** adolescent obesity, macronutrient education, cardiovascular fitness, body composition, health literacy, body image, macronutrient, educational, obesity, weight, overweight, fitness, nutrition, diet, patient education, student, school, youth, adolescent, teenager, metabolic, eating, physical activity, exercise

## Abstract

**Background:**

Obesity rates among Saudi adolescents are increasing, with regional variations highlighting the need for tailored interventions. School-based health programs in Saudi Arabia are limited and often emphasize weight and body size, potentially exacerbating body image dissatisfaction. There is limited knowledge on the feasibility of non–weight-centric educational programs in Saudi Arabia and their effects on health behaviors and body image.

**Objectives:**

This study aimed to (1) assess the prevalence of obesity using BMI-for-age *z* score (BAZ) and fat percentage among Saudi adolescents; (2) evaluate key health behaviors, cardiovascular fitness, and health literacy; and (3) assess the feasibility and impact of a low-intensity, non–weight-centric educational intervention designed to improve knowledge of macronutrients and metabolic diseases, while examining its safety on body image discrepancies.

**Methods:**

A quasi-experimental, pre-post trial with a parallel, nonequivalent control group design was conducted among 95 adolescents (58 boys and 37 girls; mean age 16.18, SD 0.53 years) from 2 public high schools in Medina City, Saudi Arabia. Participants were randomly assigned to either the weight-neutral Macronutrient + Non-Communicable Diseases Health Education group or the weight-neutral Macronutrient Health Education group. Anthropometry (BAZ and fat percentage), cardiovascular fitness, physical activity, and eating behaviors were measured at baseline. Independent *t* tests and *χ*² tests were conducted to compare group differences, and a 2-way mixed ANOVA was used to evaluate the effect of the intervention on macronutrient knowledge and body image discrepancies. A total of 69 participants completed the postintervention assessments.

**Results:**

The prevalence of overweight and obesity based on BAZ was 37.9% (36/95), while 50.5% (48/95) of participants were classified as overfat or obese based on fat percentage. Students with normal weight status were significantly more likely to have had prior exposure to health education related to metabolic diseases than students with higher weight status (*P*=.02). The intervention significantly improved macronutrient-metabolic knowledge (*F*_1,64_=23.452; *P*<.001), with a large effect size (partial η²=0.268). There was no significant change in students’ body image from pre- to postintervention (*P*=.70), supporting the safety of these weight-neutral programs. The intervention demonstrated strong feasibility, with a recruitment rate of 82.6% and a retention rate of 72.6%.

**Conclusions:**

This study reveals a high prevalence of obesity among Saudi adolescents, particularly when measured using fat percentage. The significant improvement in knowledge and the nonimpact on body image suggest that a non–weight-centric intervention can foster better health outcomes without exacerbating body image dissatisfaction. Region-specific strategies that prioritize metabolic health and macronutrient education over weight-centric messaging should be considered to address both obesity and body image concerns in adolescents.

## Introduction

Childhood obesity has become a significant public health concern globally, with projections indicating a continued upward trend in the coming decades. Obesity is expected to rise dramatically among children aged 5‐19 years, from an estimated 158 million in 2020 to 254 million by 2030 [1], representing a 60% increase over just a decade. Global estimates suggest that the overall prevalence of childhood overweight and obesity could reach 30% by 2030, with boys (34.2%) surpassing girls (27.4%) [2]. This trend is particularly alarming in middle- and high-income countries, where childhood overweight and obesity rates are projected to reach 58.3% by 2030 [2].

In Saudi Arabia, the obesity epidemic has mirrored global trends, with a marked increase in prevalence among both adults and adolescents. National estimates indicate that obesity prevalence among Saudi adolescents ranges from 22.3% to 23.5%, with some studies suggesting that nearly 35% of adolescents are overweight and 20% are obese [3-5]. Although BMI is a common tool for assessing obesity, it has limitations in distinguishing between fat and muscle mass. Notably, recent findings in Saudi Arabia highlight that BMI can underestimate obesity prevalence compared with body fat percentage [6]. For instance, while BMI data indicated an obesity prevalence of 29% (102/348) in men and 53% (314/593) in women, body fat percentage assessments revealed significantly higher rates of 83.9% (292/348) in men and 97.3% (557/593) in women [6]. This underscores the importance of using body fat percentage to provide a more accurate representation of obesity, particularly when evaluating related health risks.

Regional disparities in obesity prevalence further complicate the issue. Urbanized areas in Saudi Arabia report higher rates of obesity, influenced by factors such as physical inactivity, Westernized diets, and socioeconomic conditions [7]. Conversely, rural regions often exhibit lower obesity rates due to more traditional lifestyles that incorporate higher physical activity and different dietary patterns [7]. These regional differences emphasize the need to address physical activity behaviors and dietary habits in specific contexts. While all forms of physical activity are beneficial, current evidence indicates that vigorous-intensity exercise may be particularly effective in improving body composition, cardiorespiratory fitness, and cardiometabolic health markers in adolescents [8,9]. Despite this, there remains a gap in studies exploring the relationship between physical activity behaviors and cardiovascular fitness among adolescents in Saudi Arabia.

Adding to the complexity, decreased health literacy and higher-income levels have been linked to increased obesity rates in Saudi Arabia. This contrasts with trends observed in the United States, where lower-income levels are more commonly associated with higher obesity rates [10,11]. This highlights the crucial role of health literacy as a determinant of obesity prevalence and the importance of tailored educational interventions.

Despite the alarming trends, evidence-based interventions targeting adolescents in Saudi Arabia, particularly late adolescents in school settings, remain limited [12,13]. Schools provide an ideal environment for implementing cost-effective and sustainable obesity interventions, especially when these programs are theory-driven and tailored to meet the specific needs of the target population [14]. A review of the literature shows that only 7 school-based obesity intervention studies have targeted adolescents in Saudi Arabia, with an average age range of 12‐14 years, and none focusing on high school students [12,13,15]. Furthermore, only 3 of these studies used theory-based approaches [12], and all used weight-centric messaging.

Since 2020, more than 100 organizations worldwide, including scientific societies and academic institutions, have endorsed joint international statements to eliminate weight stigma [16]. Non–weight-centric approaches, which emphasize overall health and well-being rather than solely focusing on weight loss, have gained traction in school-based obesity interventions [17]. These programs encourage healthy behaviors and promote a positive body image without stigmatizing students, addressing the negative outcomes associated with weight stigma such as anxiety, disordered eating, and even weight gain [18,19]. However, designing obesity prevention programs that avoid weight-based stigma remains challenging, prompting calls for expanded research in non-Western cultural settings [16,20].

No study in Saudi Arabia has yet documented the development or efficacy of non–weight-centric interventions. This study focused on Medina City to examine the impact of healthy lifestyle factors on obesity rates among late adolescents and to assess the effectiveness and safety of a low-intensity, non–weight-centric, noncommunicable diseases (NCDs) educational intervention. This regional focus aims to provide insights that can inform targeted, culturally sensitive obesity prevention strategies.

In summary, this study sought to (1) identify the prevalence of obesity using both BMI-for-age *z* score (BAZ) and body fat percentage among Saudi late adolescents in Medina City; (2) characterize health-related behaviors, cardiovascular fitness, handgrip strength, and health literacy levels; and (3) evaluate the feasibility and safety of a low-intensity health educational intervention aimed at empowering adolescents with improved NCD knowledge while monitoring its effects on body image discrepancy. This research aims to contribute to the development of effective, culturally appropriate interventions that promote adolescent health without reinforcing weight stigma.

## Methods

### Design

This study used a quasi-experimental, pre-post trial with a parallel, nonequivalent control group design to assess baseline anthropometry, health-related cardiovascular fitness components, handgrip strength, physical activity, and eating behaviors, as well as the feasibility of a low-intensity, school-based educational intervention aimed at improving critical thinking about the relationship between macronutrients and NCDs among high school students, while tracking safety measures such as body image discrepancy.

### Participants

A total of 115 high school students from 2 public schools (1 for males and 1 for females) in Medina City, Saudi Arabia, were invited to participate. These schools were selected by the ministry of education’s school health department. Of the invited students, 95 (58 males and 37 females) completed anthropometry and body composition measurements. Baseline surveys were completed by 85 students (53 males and 32 females). Four classes (2 females and 2 males) were randomly assigned to 1 of 2 intervention groups: the Macronutrient+ NCDs Health Education group (n=31) or the Macronutrient Health Education group (n=38).

### Intervention: Green Apple

#### Overview

The LEAF program, an 8-session structured intervention, was developed to enhance critical thinking regarding macronutrients and their relationship to body energy and the prevention of cardiometabolic diseases. For this study, a pilot version, titled Green Apple, was conducted over two 45-minute sessions for female students and one 60-minute session for male students due to scheduling constraints. The intervention was based on best practices derived from evidence-based research into health literacy and behavior change [21]. The program was grounded in the Health Belief Model [22] and Social Cognitive Theory [23], incorporating the Transtheoretical Model for behavior change [24].

These theories provided the framework for understanding how adolescents perceive their risk of NCDs (eg, diabetes, hyperlipidemia, liver disease, stroke, and hypertension) and motivated them to adopt healthier behaviors through education, muscle building, and dietary modifications. The Green Apple program aimed to reduce chronic metabolic NCD risk by promoting visceral fat reduction and muscle building without focusing on weight loss or obesity. Participants were encouraged to follow the MyPlate dietary guidelines [25], emphasizing fiber and whole food consumption and engaging in muscle-building exercises. The program avoided topics related to obesity and sedentary behavior, instead focusing on metabolic health improvements.

#### Intervention Groups

The Macronutrient+ NCDs Health Education group included 3 educational topics covering nutrition and metabolic diseases (eg, diabetes and cardiovascular disease), highlighting the impact of macronutrition on metabolic health.

The Macronutrient Health Education group included 2 educational topics focused solely on healthy nutrition principles, dietary guidelines, food groups, and balanced eating.

Both groups received identical macronutrient content.

### Measurements

#### Demographics

Baseline demographic data included age, self-reported weight and height, parental education levels, and monthly family income. Parental education was categorized as high (college degree or above) or low (high school degree or below).

#### Anthropometry

Body weight and height was measured using a portable digital scale (Omron BF511) to the nearest 100 g and a stadiometer to the nearest 0.1 cm. BMI was calculated as weight (kg) divided by height squared (m²), and BAZs were classified using the World Health Organization 2007 Growth Reference for children and adolescents aged 5‐19 years [26]. The BAZ categories were thinness (−3≤ BAZ <−2), normal weight (−2≤ BAZ ≤+1), overweight (+1< BAZ ≤+2), and obesity (BAZ >+2).

Fat percentage was assessed using a bioimpedance analyzer (Omron BF511). Classification was based on McCarthy’s age- and sex-specific fat percentile references, with the 2nd, 85th, and 95th percentiles defining underfat, overfat, and obese, respectively [27].

#### Cardiovascular Fitness

The Queen’s College Step Test, a submaximal exercise test, was used to estimate the maximal oxygen consumption (VO_2_max). Participants stepped on a 30.5- cm box at a set rate for 3 minutes [28]. The 30.5-cm step height was chosen for its suitability for adolescents [28]. VO_2_max estimation: The pulse rate was measured postexercise using a pulse oximeter, following McArdle’s protocol [29]. The equations for estimating VO_2_max were as follows: girls: 65.81 − (0.1847 × pulse rate) and boys: 111.33 − (0.42× pulse rate) [28].

#### Handgrip Strength

Handgrip strength was measured using a Takei Kiki Kogyo dynamometer, and the highest value from 2 trials for the dominant hand was recorded. Classifications followed age- and gender-specific percentiles [30].

#### Physical Activity

Physical activity was assessed using the Arab Teen Lifestyle Study physical activity questionnaire subscale [31], calculating total weekly energy expenditure in metabolic equivalent tasks (METs), with vigorous activities (6 METs) and moderate activities (4 METs) expressed in minutes per week.

#### Eating Habits

Eating habits were evaluated using the Arab Teen Lifestyle Study eating habits subscale, which measured positive eating habits (eg, fruit or vegetable intake) and negative eating habits (eg, sugary drinks or fast food) based on weekly frequency [31], with a total scores ranging from 0 to 28 for positive habits and 0 to 35 for negative habits.

#### Sedentary Behavior

Sedentary behavior was measured using the Arabic Sedentary Behavior Questionnaire (SBQ) for weekdays [32]. The questionnaire included 9 items, and a total sitting time was averaged over 5 weekdays. Sitting ≥7 hours per day was considered highly sedentary [33].

#### Exposure to NCDs Health Education

Exposure to health education on NCDs was assessed using 4 yes or no questions regarding prior education on chronic diseases. A total score (0‐4) was calculated.

#### Macronutrient-NCDs Knowledge

Macronutrient and NCDs knowledge was measured using an 18-item true-or-false quiz developed based on the Green Apple content. A total score (0‐18) was analyzed, with an urgent need for intervention indicated if 70% or fewer answered correctly, a considered need at 71%‐89%, and no need at 90% or more [34].

#### Body Image Discrepancy

Body image discrepancy was assessed using 4 body size silhouettes [35]. Participants selected their ideal and perceived current body images, with discrepancies indicating body image concerns. A negative score represented a drive for thinness, while a positive score reflected a drive for increased body weight [36].

### Ethical Considerations

The study protocol was approved by the Shaqra University ethics committee (ERC_SU_20230005). Written informed consent was obtained from parents, and student participation was voluntary. To ensure privacy and confidentiality, participant data were anonymized using unique identifiers, and data access was limited to authorized personnel. Baseline assessments were conducted prior to the intervention. The intervention was delivered on different days for males and females due to scheduling constraints. Postintervention assessments for macronutrient-NCDs knowledge and body image discrepancy were conducted, and participants received a key chain medley as a token of appreciation for their involvement.

### Statistical Analysis

#### Overview

Descriptive statistics (means, SDs, and frequencies) were calculated for all variables. Independent *t* tests were conducted to compare males and females and students with and with no overweight or obesity on continuous variables, while *χ*² tests were used for categorical variables. A 2-way mixed ANOVA design was used, with 1 between-subjects factor (intervention type: macronutrient-NCDs vs macronutrient) and 2 within-subjects factors (time: pre-test and post-test) for 2 dependent variables: macronutrient-NCDs knowledge and body image discrepancy. Gender was included as an additional between-subjects variable to assess potential interactions with time and intervention type.

Statistical significance was set at *P*<.05. Sample size calculations using G*Power 3.1 indicated a required sample size of 34 participants to detect medium effects (*f*=0.25) with 80% power. Although an initial plan accounted for a 30% attrition rate, the final sample size exceeded the minimum requirement for detecting a medium effect size with 80% power, ensuring sufficient study power.

A 2-way mixed ANOVA was conducted to evaluate the effect of the Green Apple intervention on students’ macronutrient-NCDs knowledge scores before and after the intervention and to determine potential gender interaction effects, given the significant difference in mean scores between males and females at preassessment.

Two borderline outliers with studentized residuals of −3.02 and −3.03 were identified but retained in the analysis. The data were normally distributed, as assessed by the Shapiro–Wilk test (*P*>.05). Homogeneity of variances (*P*>.05) and covariances (*P*>.001) were confirmed by Levene test and Box M test, respectively, except for postintervention body image discrepancy, which required Welch ANOVA.

#### Missing Data

A missing data analysis was conducted, revealing that 4% of data were missing across all items, with a maximum of 7% missing for certain variables (eg, “minutes’ walk per day,” “number of stairs per day,” and “SBQ-listening to music, using computer, crafting”). Little MCAR test was applied to assess the randomness of the missing data, yielding a nonsignificant result (*P*>.05), indicating that the data were missing completely at random [37]. Expectation-Maximization was used to impute missing data for variables with 5%‐7% missing values. For variables with <5% missing data, series mean and series median imputation methods were used.

Two items from the physical activity scale (“regular dancing” and “house cleaning”) were excluded due to cultural factors that led to inconsistent responses between male and female participants. This decision was informed by a review of similar studies conducted in the region [38,39]. Furthermore, family income was excluded from analysis because female students were often unaware of this information, which is considered sensitive, and younger students may not accurately understand their family’s financial status [40]. The high absenteeism rate among female students during the intervention (approximately 28%) also contributed to some missing data, consistent with previously reported absenteeism rates in Saudi schools [41].

## Results

### Baseline Characteristics and Prevalence of Overweight and Obesity

Table 1 shows the baseline characteristics of the 95 participants (37 girls and 58 boys). The mean age of the total sample was 16.18 (SD 0.53) years, with girls having a significantly higher mean age than boys (*P*<.001). The mean BAZ score for the total sample was 0.44 (SD 1.42), with a significant sex difference (*P*=.03). Furthermore, girls had a higher mean fat percentage than boys (28.58% vs 22.65%), but this difference was not statistically significant (*P*=.37). The combined prevalence of overweight and obesity based on the BAZ classification was 37.9% (36/95), with 16.8% (16/95) of participants classified as overweight and 21.1% (20/95) of participants classified as obese. When considering fat percentage, the combined prevalence of overfat and obesity was 50.5% (48/95), with 11.6% (11/95) of participants classified as overfat and 38.9% (37/95) of participants classified as obese. For the BAZ and fat percentage percentile categories, no significant sex differences were observed. Table 2 shows descriptive statistics for the distribution of the participants’ weight status (normal BAZ and high BAZ) across different levels. These results suggest that there was no statistically significant relationship between parental education level (both mother and father) and the participants’ weight category.

**Table 1.** Descriptive statistics based on sex (mean [SD] or fat percentage).

Variables	All (N=95)	Girls (n=37)	Boys (n=58)	Sex (significance) difference^a^
Age (years), mean (SD)	16.18 (0.53)	16.38 (0.64)	16.05 (0.39)	<.001
BMI (kg/m^2^), mean (SD)	23.46 (6.07)	23.25 (5.24)	23.59 (6.59)	.05
BAZ^b^, mean (SD)	0.44 (1.68)	0.44 (1.42)	0.44 (1.84)	.03
Fat percentage, mean (SD)	24.96 (9.54)	28.58 (8.62)	22.65±9.44	.37
**BAZ percentile, n (%)**				.39
Underweight	7 (7.4)	2 (5.4)	5 (8.6)	
Normal weight	52 (54.7)	22 (59.5)	30 (51.7)	
Overweight	16 (16.8)	8 (21.6)	8 (13.8)	
Obese	20 (21.1)	5 (13.5)	15 (25.9)	
**Fat percentage percentile, n (%)**				.44
Underfat	5 (5.3)	3 (8.1)	2 (3.4)	
Normal	42 (44.2)	16 (43.2)	26 (44.8)	
Overfat	11 (11.6)	6 (16.2)	5 (8.6)	
Obese	37 (38.9)	12 (32.4)	25 (43.1)	
Exposure to metabolic NCDs^c^ education, mean (SD)^d^	3.19 (1.006)	3.72 (0.523)	2.87 (1.093)	<.001
Macronutrient-metabolic NCDs knowledge, mean (SD)^d^	10.64 (3.638)	12.13 (2.472)	9.74 (3.943)	.50
Body image discrepancy, mean (SD)^e^	−0.36 (0.94)	−0.69 (0.74)	−0.13 (1.00)	.005

a Independent *t* tests were conducted to compare differences between the 2 groups. Two-sided *P* values are reported when no significant difference was found between the groups. One-sided *P* values are reported when the 1-directional hypothesis was supported.

b BAZ: BMI-for-age *z* score.

c NCD: noncommunicable disease.

d The n values for these data are as follows: All (n=85); Girls (n=32); Boys (n=53).

e The n values for these data are as follows: All (n=78); Girls (n=32); Boys (n=46).

**Table 2.** Weight category descriptive statistics (mean [SD] or fat percentage).

	All	Normal BAZ^a^	High BAZ	Significance^b^
**Mother’s education, n (%)**				.99
Low education level	28 (34.6)	18 (34.6)	10 (34.5)	
High education level	53 (65.4)	34 (65.5)	19 (65.5)	
**Father’s education, n (%)**				.07
Low education level	18 (21.7)	8 (15.4)	10 (32.3)	
High education level	65 (78.3)	44 (84.6)	21 (67.7)	
Fat percentage, mean (SD)	24.96 (9.54)	19.53 (6.10)	33.85 (7.16)	<.001
VO_2_max (mL/kg/min), mean (SD)	53.33 (14.63)	53.68 (14.72)	52.77 (14.67)	.86
VO_2_max boys (mL/kg/min), mean (SD)	62.65 (10.29)	63.43 (10.69)	61.46 (9.77)	.81
VO_2_max girls (mL/kg/min), mean (SD)	37.90 (2.87)	38.84 (1.83)	36.10 (3.65)	.014
Handgrip strength max score (kg), mean (SD)	24.49 (7.85)	24.81 (8.81)	23.96 (6.04)	.15
**Handgrip strength percentile, n (%)**				.67
Low: ≤20	52 (54.7)	34 (57.6)	18 (50)	
Normal: 21‐80	35 (36.8)	21 (35.6)	14 (38.9)	
High: >80	8 (8.4)	4 (6.8)	3 (11.1)	
**Health-related behaviors based on weight status, mean (SD)**				
Vigorous-intensity physical activity (METs^c^ min/wk)	2038.91 (2560.25)	2360.92 (2914.76)	1505.56 (1743.45)	.047
Moderate-intensity physical activity (METs min/wk)	1038.90 (1219.13)	1009.073 (1174.13)	1088.286 (1307.99)	.39
Sedentary behavior per day during the week (h/d)	7.28 (3.93)	7.741 (3.11)	7.5064 (3.98)	.34
Positive eating behavior	16.43 (6.30)	16.838 (5.98)	15.7188 (6.84)	.22
Negative eating behavior	15.11 (6.62)	15.160 (6.22)	15.022 (7.34)	.46
Exposure to metabolic NCDs^d^ health education topics	3.19 (1.01)	3.36 (0.76)	2.91 (1.28)	.02
Macronutrient-metabolic NCDs knowledge	10.64 (3.64)	10.90 (3.10)	10.13 (4.39)	.16
**Need for educational intervention assessment, n (%)**				.92
Urgent need	59 (69.4)	37 (69.8)	22 (68.8)	
Considered need	26 (30.6)	16 (30.2)	10 (31.3)	
No need	0 (0)	0 (0)	0 (0)	

a BAZ: BMI-for-age *z* score.

b Independent *t* tests were conducted to compare differences between the 2 groups. Two-sided *P* values are reported when no significant difference was found between the groups. One-sided *P* values are reported when the 1-directional hypothesis was supported.

c MET: metabolic equivalent task.

d NCD: noncommunicable disease.

### Cardiovascular Fitness and Handgrip Strength

Overall cardiovascular fitness levels, as measured using VO_2_max, did not significantly differ between individuals in the normal and high body weight categories. The boys in the normal BAZ group had a slightly higher VO_2_max (63.43 mL/kg/min, SD 10.69) than the boys in the high BAZ group (61.46 mL/kg/min, SD 9.77). However, this difference was not statistically significant (*P*=.81). There was a notable and statistically significant difference between the girls in the normal BAZ group (38.84 mL/kg/min, SD 1.83) and those in the high BAZ group (36.10 mL/kg/min, SD 3.65), with a *P* value of .014. This indicates that girls with overweight or obesity had significantly lower cardiovascular fitness levels than their peers in the normal group, suggesting a potential negative impact of higher body weight on cardiovascular fitness among girls. However, the distribution of handgrip strength categories did not show a statistically significant association with the BAZ classification (*P*=.67) (Table 2).

### Health-Related Behaviors Based on Weight Status

#### Activity Behavior

Participants with a high BAZ had lower levels of METs minutes per week from vigorous-intensity activity (1505.56, SD 1743.45) than those with a normal BAZ (2360.92, SD 2914.76), with a significant difference (*P*=.047). No significant differences were observed in METs from moderate-intensity activity (*P*=.39).

#### Sedentary Behavior

The difference in sedentary behavior between the 2 groups was not statistically significant (*P*=.68), indicating similar levels of sedentary time regardless of weight status.

#### Eating Behavior

Positive eating behaviors were marginally lower in the high BAZ group (15.72, SD 6.84) than in the normal BAZ group (16.84, SD 5.98), but the difference was not statistically significant (*P*=.23). Furthermore, negative eating behaviors showed no significant differences between the groups (*P*=.93).

### Macronutrient-Metabolic NCDs Knowledge and Need for Intervention

The participants with a normal BAZ reported higher exposure to metabolic NCDs education (3.36, SD 0.76) than those with a high BAZ (2.91, SD 1.28), with a significant difference (*P*=.02). However, no significant differences were observed in the macronutrient-metabolic NCDs knowledge scores between the 2 groups (*P*=.16). The majority of the participants were classified as having an urgent need for an educational intervention (59/85, 69.4%), and 30.3% (26/85) were classified as having a need for an intervention, with no statistically significant distribution between the 2 groups (*P*=.92).

### The Green Apple Intervention Effectiveness

The final sample consisted of 69 participants, with 23 females (intervention=13, control=10) and 46 males (intervention=18, control=28).

#### Effect on Macronutrient-Metabolic NCDs Knowledge

There was a significant main effect of time on students’ macronutrient-metabolic NCDs knowledge (*F*_1,58_=23.263; *P*<.001), with a large effect size (partial η²=0.286). This indicates that knowledge significantly improved from pre- to postintervention, and the magnitude of the change was substantial. Also, a significant main effect was found for intervention type (*F*_1,58_=19.756; *P*<.001), with a large effect size (partial η²=0.254). The Macronutrient + NCDs intervention had a greater effect on improving knowledge (Table 3). The interaction between gender and intervention type was nonsignificant (*F*_1,58_=0.002; *P*=.99), with a very small effect size (partial η²=0.00), indicating that the effect of the interventions on knowledge was consistent across genders.

**Table 3.** Changes in macronutrient-noncommunicable disease (NCD) knowledge and body image discrepancy across intervention periods.

	Baseline, mean (SE)	95% CI		Follow-up, mean (SE)	95% CI
**Effect on macronutrient-metabolic NCDs knowledge**					
Nutrition+ NCD	11.16 (0.56)	10.03 to 12.29		13.39 (0.42)	12.55 to 14.24
Nutrition	11.53 (0.55)	10.45 to 12.63		13.04 (0.41)	12.22 to 13.86
**Effect on body image discrepancy**					
Nutrition + NCD	−0.38 (0.17)	−0.72 to −0.03		−0.53 (0.19)	−0.90 to −0.16
Nutrition	−0.50 (0.167)	−0.84 to −0.17		−0.41 (0.18)	−0.77 to −0.05

#### Body Image Discrepancy

The effect of time on body image was nonsignificant (*F*_1,58_=0.150; *P*=.70), with a small effect size (partial η²=0.003), suggesting no significant change in students’ body image from pre- to postintervention. Similarly, the interaction between gender and intervention type was nonsignificant for body image (*F*_1,58_=0.182; *P*=.73), with a very small effect size (partial η²=0.002), meaning that the interventions had similar effects on body image for both males and females. The effect of gender on body image was marginally significant (*F*_1,58_=6.157; *P*=.052), with a medium effect size (partial η²=0.064), suggesting a slight difference in body image perception between males and females. However, Welch ANOVA was used due to unequal variances between males and females on postintervention body image discrepancy, and the findings suggest that gender does play a role in postintervention body image, with a statistically significant difference detected between males and females (Welch *F*_1,66.977_=5.385; *P*=.02). This suggests that after accounting for unequal variances (based on Levene test), gender does have a significant effect on postintervention body image.

### The Green Apple Intervention Feasibility

The high recruitment rate indicates strong initial interest and willingness to participate in the intervention, with 82.6% (95/115) of invited students enrolling in the study. Nearly 90% (85/95) of the recruited participants were engaged enough to provide comprehensive baseline data. The overall retention rate, based on completion of the pre- and postintervention questionnaires, was 72.6% (69/95). This indicates that a significant majority of the participants remained engaged with the study through to its conclusion, although there was a drop-off of 27.4% (26/95) from baseline to the postintervention phase. This dropout rate highlights some challenges in maintaining participant engagement over the study period. The retention rate was notably higher among male participants (46/58, 79.3%) than among female participants (23/37, 62.2%). This disparity was related to high absenteeism in the female school. Furthermore, one of the key challenges encountered was scheduling conflicts, particularly in the male school. These constraints impacted the intervention delivery, reducing the planned 2 sessions over 2 weeks to a single session on 1 day.

## Discussion

### Principal Findings

This study aimed to assess the prevalence of obesity among late adolescents in Medina City, Saudi Arabia, using both BAZ and fat percentage, and to evaluate the feasibility and effectiveness of a low-intensity, non–weight-centric educational intervention focused on macronutrient-metabolic disease knowledge. The findings indicate a high prevalence of obesity in this population, surpassing national averages. The Green Apple intervention effectively improved students’ macronutrient knowledge without negatively affecting body image discrepancy, demonstrating its potential as a health education tool.

The prevalence of overweight and obesity based on the BAZ classification was 37.9% (36/95), with 21.1% (20/95) classified as obese. When using fat percentage, the prevalence was even higher at 50.5% (48/95), highlighting that BMI alone may underestimate obesity rates. This aligns with regional data, indicating that adolescent obesity trends in Medina reflect broader patterns observed in the western region of Saudi Arabia [7]. The effectiveness of the Green Apple intervention in significantly improving macronutrient-metabolic NCDs knowledge without impacting body image discrepancy underscores the potential for brief educational programs to enhance adolescents’ understanding of nutrition and health.

Our study’s obesity prevalence rates are consistent with findings from prior research in the western region of Saudi Arabia. For instance, a 2019 study reported a 35.3% (121/342) obesity prevalence among university students in Medina [42], indicating a persistent trend across educational levels. A 2021 study also found a similar overweight and obesity prevalence of 38.5% (44,826/116,656) among participants aged 17‐25 years across different Saudi regions [43]. The higher prevalence of obesity in males (15/58, 25.9%) than in females (5/37, 13.5%) aligns with national data showing higher obesity rates in male adolescents [10].

The use of fat percentage as an additional measure provided a more nuanced understanding, revealing an obesity rate nearly double that of the BAZ classification. This supports prior research suggesting that fat percentage often results in a higher reported prevalence of obesity compared with BMI, sometimes by 1.5-3 times [44-46]. BMI fails to distinguish between muscle and fat mass, making fat percentage a more accurate indicator of obesity [45,46].

The study also identified a significant difference in VO_2_max scores between the normal and high BAZ groups among females, with the normal group demonstrating better cardiovascular fitness. This finding suggests an inverse relationship between BAZ and cardiovascular health, emphasizing the importance of maintaining a normal BAZ for better fitness. Similar studies have shown a negative relationship between BMI and cardiorespiratory fitness in Saudi youth aged 8‐15 years [47]. Furthermore, while the normal BAZ group had higher handgrip strength, there was no significant difference in percentiles between groups, indicating that low muscle strength is common among adolescents regardless of BMI. Previous studies have also reported low muscle strength among Saudi male adolescents aged 17 years [48] and no significant association between BMI and strength in college-aged females [49]. This study is the first to report age- and sex-specific handgrip strength percentiles for this population, an essential aspect for future research [30].

Also, the lack of significant differences in dietary behaviors between weight groups suggests that suboptimal eating habits are widespread. Both groups showed low fruit and vegetable intake and high consumption of sugary drinks, aligning with global patterns [50]. Sedentary behavior was similar between groups, reinforcing the need for targeted physical activity interventions. Our finding that vigorous physical activity correlates with lower obesity rates aligns with the Green Apple program’s emphasis on high-intensity activity for metabolic health [51]. Current evidence suggests that vigorous intensity exercise may be particularly effective for improving body composition and health outcomes in adolescents [8,9]. These modalities also show promise for improving adherence. However, proper supervision and safety considerations are important, especially for higher-intensity activities and more research is needed to determine optimal exercise prescriptions and implementation strategies for this age group [8,9].

Our findings further revealed significantly lower exposure to NCD education among students with overweight or obese status compared with those with normal weight, and male students reported lower exposure than females. This could be linked to lower NCD knowledge among male teachers, as suggested by a recent study in Saudi Arabia [52]. The lower awareness about NCDs among young adults highlights the need for targeted interventions to increase education and awareness [53,54]. Early education can lead to better outcomes by promoting preventive measures [54,55].

The efficacy of low-intensity, non–weight-centric educational interventions in improving knowledge is crucial for preventing metabolic NCDs. By focusing on macronutrient education without emphasizing weight loss, the Green Apple program demonstrates a promising approach to improving metabolic health in adolescents without affecting body image. Research on educational interventions in Saudi Arabia and Gulf Cooperation Councilcountries is limited, with existing studies primarily focusing on obesity awareness and metabolic NCDs such as diabetes [12,56]. Our study contributes to this field by showing that even low-intensity interventions targeting macronutrient and NCD knowledge can enhance health literacy. Importantly, this study targeted late adolescents, addressing a significant gap as previous research has mainly focused on younger adolescents (aged 12‐14 years) [12,56]. The Green Apple program, as a pilot derived from the larger LEAF program, suggests a scalable model that integrates educational and behavioral strategies to promote sustainable health improvements by focusing on visceral fat reduction and muscle mass development.

The significant improvement in students’ knowledge aligns with recent literature highlighting the importance of early NCD education [57,58]. The observed large effect size (partial η²=0.286) further supports the intervention’s effectiveness. Contextualizing nutritional information to metabolic health may enhance learning by linking knowledge to practical outcomes. Also, the nonsignificant interaction between gender and intervention type indicates that the content was equally effective for both male and female students, which is significant in a cultural context where gender-specific education is common [59]. This suggests that balanced content can be effective across genders. Finally, the nonsignificant effect on body image supports literature advocating for weight-neutral interventions to avoid adverse body image outcomes [60]. However, the significant postintervention body image difference between genders highlights the need for considering gender-specific perceptions in future program designs [61].

### Limitations and Recommendations

Despite the positive outcomes, challenges such as scheduling and gender-specific retention highlight the need for flexible strategies. Limitations include the relatively small, single-region sample size, which may limit generalizability, and the short intervention period, which may not capture long-term effects. Self-reported data could introduce bias. Future research should involve larger, more diverse samples and longer interventions and consider digital family-based education methods, such as webinars and web-based workshops, to enhance parental involvement [62-64]. Engaging parents in school health education could yield better adolescent health outcomes [63,64].

### Conclusions

This study underscores the high prevalence of obesity among Saudi adolescents and highlights the potential of low-intensity educational interventions to improve macronutrient knowledge. By emphasizing metabolic health and muscle building, the Green Apple program offers a promising non–weight-centric strategy for preventing chronic metabolic diseases. Future programs should focus on high-intensity physical activity and neutral weight messaging to address obesity and mental health comprehensively.

## Abbreviations

BAZ: BMI-for-age *z* score
MET: metabolic equivalent task
NCD: noncommunicable disease
SBQ: Sedentary Behavior Questionnaire
VO_2_max: maximal oxygen consumption
